# Staphylococcal SplA and SplB Serine Protease Allelic Variants Exhibit Different Substrate Specificities

**DOI:** 10.1002/cbic.202500816

**Published:** 2025-12-22

**Authors:** Felix L. Glinka, Katharina Mehnert, Lena Koch, Ole Schmöker, Leif Steil, Christian Hentschker, Uwe Völker, Thor B. Johannesen, Dominique Böttcher, Michael Lammers, Barbara M. Bröker, Uwe T. Bornscheuer

**Affiliations:** ^1^ Dept. of Biotechnology & Enzyme Catalysis Institute of Biochemistry University of Greifswald 17489 Greifswald Germany; ^2^ Dept. of Synthetic and Structural Biochemistry Institute of Biochemistry University of Greifswald 17489 Greifswald Germany; ^3^ Dept. of Functional Genomics Interfaculty Institute for Genetics and Functional Genomics University of Greifswald 17475 Greifswald Germany; ^4^ Dept. of Sequencing and Bioinformatics Statens Serum Institut 2300 Copenhagen Denmark; ^5^ Institute of Immunology University Medicine Greifswald 17489 Greifswald Germany

**Keywords:** protein degradation, *S* *taphylococcus aureus*, serine protease‐like, SplA, SplB, substrate specificity

## Abstract

*Staphylococcus aureus* is an opportunistic pathogen that is persistently colonizing nearly 30% of the human population and can cause life‐threatening infections. *S. aureus* secretes a variety of virulence factors, such as a set of extracellular serine protease‐like proteins (Spls). Spls are expressed by most clinical isolates of *S. aureus*, but their pathophysiological substrates and role during infection are largely unknown. Pathogens use allelic variation of virulence factors to allow an adaption to different host cells and their defense mechanisms. The differences of these variants are marginally characterized so far. Here, we performed a biochemical characterization of selected allelic variants of the *S. aureus* SplA and SplB. Our data suggests different variants show differences in their stability, enzymatic activity, and substrate specificity. For the recently identified Spl target proteins RickULP and SseL, different cleavage patterns were observed, upon treatment with different Spl allelic variants. One SplB variant strongly differed in its substrate recognition at the P3/P4 position, more closely resembling SplE than SplB wildtype in its substrate selectivity. Our data provide valuable insights into the evolution of bacterial virulence factors and highlight the importance of including allelic variation of virulence factors to fully understand their role in host–pathogen interaction.

## Graphical abstract

A biochemical characterization of *Staphylococcus aureus* serine proteases SplA and SplB allelic variants (recombinantly produced in *E. coli* and purified) reveals that allelic variation adds an extra layer of complexity to the functioning of virulence factors in *S. aureus* colonization and infection. This highlights the importance of considering allelic variation in the study of host‐pathogen interactions.

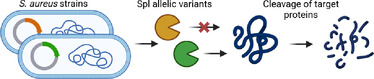

## 1 Introduction

*Staphylococcus aureus* (*S. aureus*) is a Gram‐positive opportunistic human pathogen and a leading cause of infections. On the one hand, ≈30% of the human population is persistently colonized without visible adverse effects. On the other hand, the bacterium is a major cause of community‐acquired and nosocomial infections that can result in life‐threatening conditions like sepsis, osteomyelitis, toxic shock syndrome, and pneumonia.^[^ ^1^ ^,^ ^2^ ^]^ *S. aureus* has the ability to persist intracellularly and can therefore evade recognition and elimination by the host's immune system or antibiotic therapy, leading to recurrent infections.^[^ ^3^ ^,^ ^4^ ^]^ Additionally, the occurrence of multi‐drug resistant strains makes targeted and successful antibiotic treatment increasingly difficult.^[^ ^1^ ^,^ ^5^ ^]^

*S. aureus* secrets a variety of virulence factors, including numerous proteases.^[^ ^2^ ^]^ Among them is a set of extracellular serine proteases: the serine protease‐like proteins (Spls). The *spl* operon is encoded on the *ν*Sa*β* pathogenicity island alongside with other known *S. aureus* virulence factors, suggesting an important role during bacterial infection.^[^ ^6^ ^,^ ^7^ ^]^ The operon is composed of six genes encoding the proteases SplA to SplF.^[^ ^8^ ^,^ ^9^ ^]^ The *spl* genes are found in the majority of *S. aureus* strains, but the composition of the *spl* operon varies. A study comprising of 96 nasal *S. aureus* isolates showed that 82% carried at least one *spl* gene and ≈19% of the isolates carried all six *spl* genes.^[^ ^10^ ^]^ The broad distribution of these genes across different *S. aureus* strains indicates an important function of the Spls. When the *spl* operon was reintroduced into *S. aureus* strains devoid of other extracellular proteases, its virulence increased dramatically in murine bloodstream infection.^[^ ^11^ ^]^ Although the Spls were discovered in 1996,^[^ ^8^ ^]^ their pathophysiological substrates and biological functions during infection remain largely unknown.

SplB was previously shown to cleave several central human complement proteins, thereby inhibiting all three complement pathways (classical, lectin and alternative pathway) and blocking opsonophagocytosis—a crucial part of the immune system's response to bacterial infection.^[^ ^10^ ^]^ In a previous study, we identified several deubiquitinating enzymes (DUBs) involved in human immune signaling pathways as targets of SplA and SplB.^[^ ^12^ ^]^ These findings underline, that Spls play a major role in the pathogenicity of *S. aureus*.

Allelic variation—variants of a gene where the DNA sequence differs between two or more bacterial strains of the same species—plays a crucial role in enabling bacterial adaptation to different host cells and/or environmental conditions and it contributes to host specificity and drug resistance.^[^ ^13^ ^]^ The species *S. aureus* exhibits significant allelic variation, particularly in genes related to virulence and antibiotic resistance.^[^ ^13^, ^14^, ^15^ ^]^ These variations influence the molecular mechanisms underlying infection of host cells that influence disease development and affect the responsiveness of host organisms to an anti‐infective treatment. Allelic variation of secreted serine proteases can also determine host range plasticity in bacteria.^[^ ^16^ ^]^ Investigating the impact of allelic variation of the staphylococcal Spl proteases on their substrate specificity, etc., could therefore help to understand the Spl‐mediated pathology during *S. aureus* infection.

Here, we aimed at a detailed biochemical characterization and identification of cleavage specificities of major staphylococcal SplA and SplB allelic variants. Our data show that allelic variants of Spls differ in their protein stability and proteolytic activity as well as substrate selectivity and/or cleavage motifs. These data provide valuable information on the evolution of virulence factors and the adjustment of bacterial pathogens to their host cells by using a repertoire of allelic variants of virulence factors.

## 2 Results

### 2.1 Selection of SplA and SplB Allelic Variants for Biochemical Characterization

The sequences of the allelic variants of SplA and SplB studied in this work were obtained from bacteria in patient blood samples that were reported in the National Center for Biotechnology Information (NCBI) Reference Sequence Database (RefSeq).^[^ ^17^ ^]^ All sequences originate from naturally occurring *S. aureus* strains. The dataset contains 9230 variants (unique FASTA IDs) of the *spl* operon, which differ in composition or the DNA sequences of the individual *splA‐F* genes located in the operon. The *spl* genes are natively expressed by *S. aureus* with a 35–36 amino acid signal peptide which is cleaved off precisely during secretion, activating the Spls.^[^ ^9^ ^,^ ^18^ ^,^ ^19^ ^]^ Excluding variations in the signal peptide, the *spl* allelic variants encode for around 100–150 unique amino acid sequences of each mature SplA‐F protein. SplA and SplB are both encoded by ≈90% of the *spl* operons, and they each have two predominant amino acid sequences that are present in 65‐80% of the *spl* operon variants.

**Figure 1:**
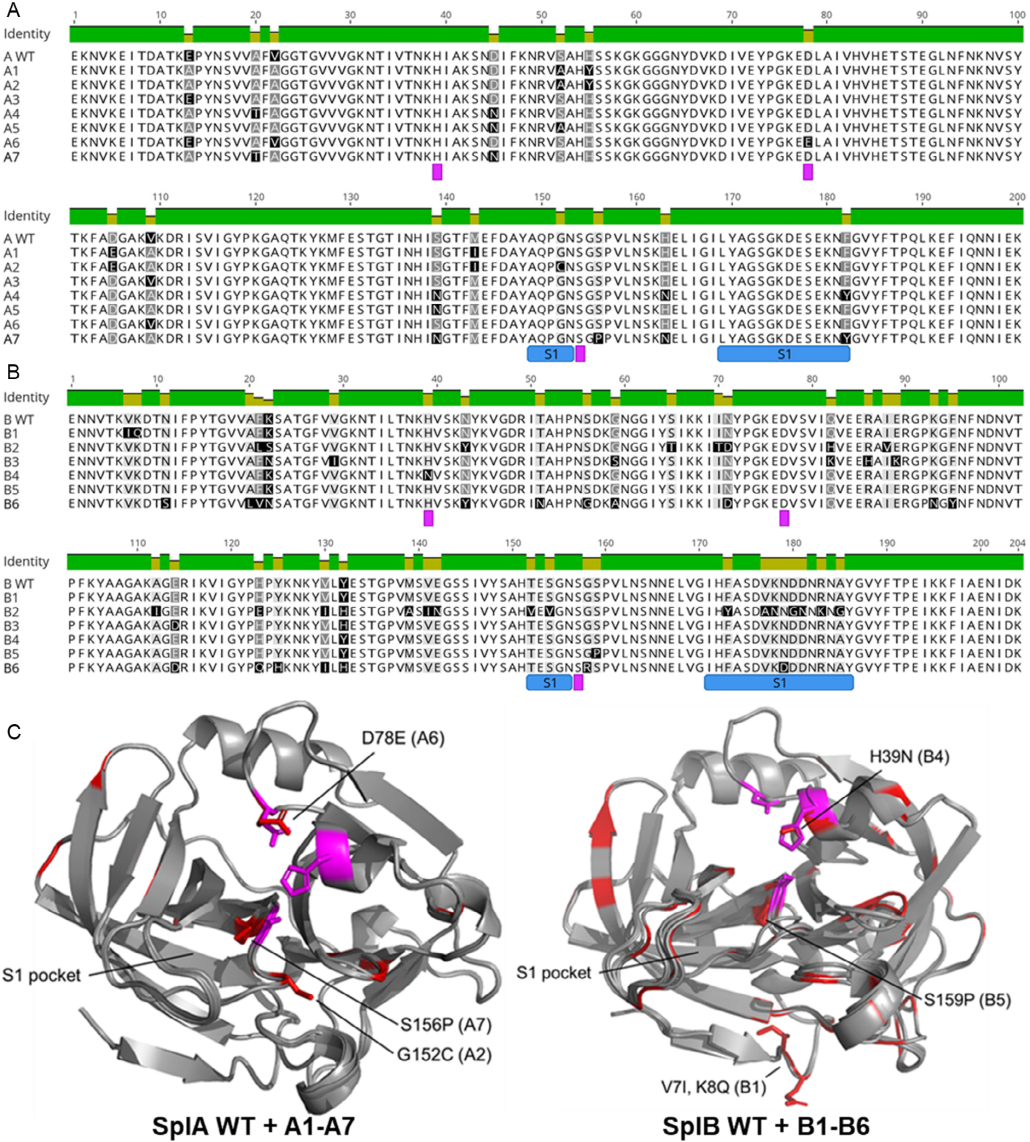
Overview of selected SplA and SplB allelic variants. Multiple sequence alignments of A) SplA and B) SplB allelic variants. The catalytic triad (Ser‐His‐Asp) is annotated with magenta squares and the loop structures that build the S1 substrate specificity pocket are annotated in blue squares below the alignments. C) Structural alignments of the SplA and SplB allelic variants (AlphaFold3 predicted structured) with RMSD of 0.1–0.2 Å for SplA variants and 0.2–0.7 Å for SplB variants. The catalytic triad is highlighted in magenta and variant sites are marked in red. The S1 pocket and several variation sites of special interest are marked with the specific amino acid exchange and the corresponding Spl variant.

Our previous work was focused on the SplA and SplB variants of *S. aureus* strain USA300, which cover 39% and 35% of the reported unique protein sequences, respectively.^[^ ^10^ ^,^ ^12^ ^]^ For simplicity, we call these variants wildtype (WT) in this report (sequences are provided in the Supporting Information). For this study, an additional set of SplA and SplB allelic variants was selected for biochemical characterization and comparison with the WT variants (A1‐A7 and B1‐B6, Table S1, Supporting Information). The set includes the most abundant Spl variants (number of *spl* operons encoding for a given unique mature Spl sequence) as well as variants containing mutations in and around the catalytic center or in other highly conserved sequence motifs (**Figure** **1**). For example, some variants carry a mutation in the GN**S**GSP motif which contains the catalytic serine (marked in bold) and is highly conserved among the Spls and their allelic variants. This motif is a variation of the highly conserved GD**S**GGP motif of the peptidase family S1 (chymotrypsin‐like), to which the Spls belong, and it is important for the correct folding and functioning of serine proteases.^[^ ^20^ ^]^ Three of the SplB variants (B2, B3 and B7) were selected, because they contain mutations which impacted SplB activity in another study.^[^ ^21^ ^]^ Most selected Spl variants contain 1–9 mutation sites compared to the respective WT (99‐95% sequence identity), while the variants B2 and B6 have 24 and 16 mutations with 88 and 91% sequence identity, respectively (Figure 1). In comparison, SplA‐F share a sequence identity of 44‐95% among each other, with the SplA and SplB WTs having a sequence homology of 48% (Table S2, Supporting Information). All Spls were recombinantly produced in *E. coli* and purified by affinity chromatography before biochemical characterization.

### 2.2 Thermal Stability Characterization of SplA and SplB Allelic Variants

**Figure 2:**
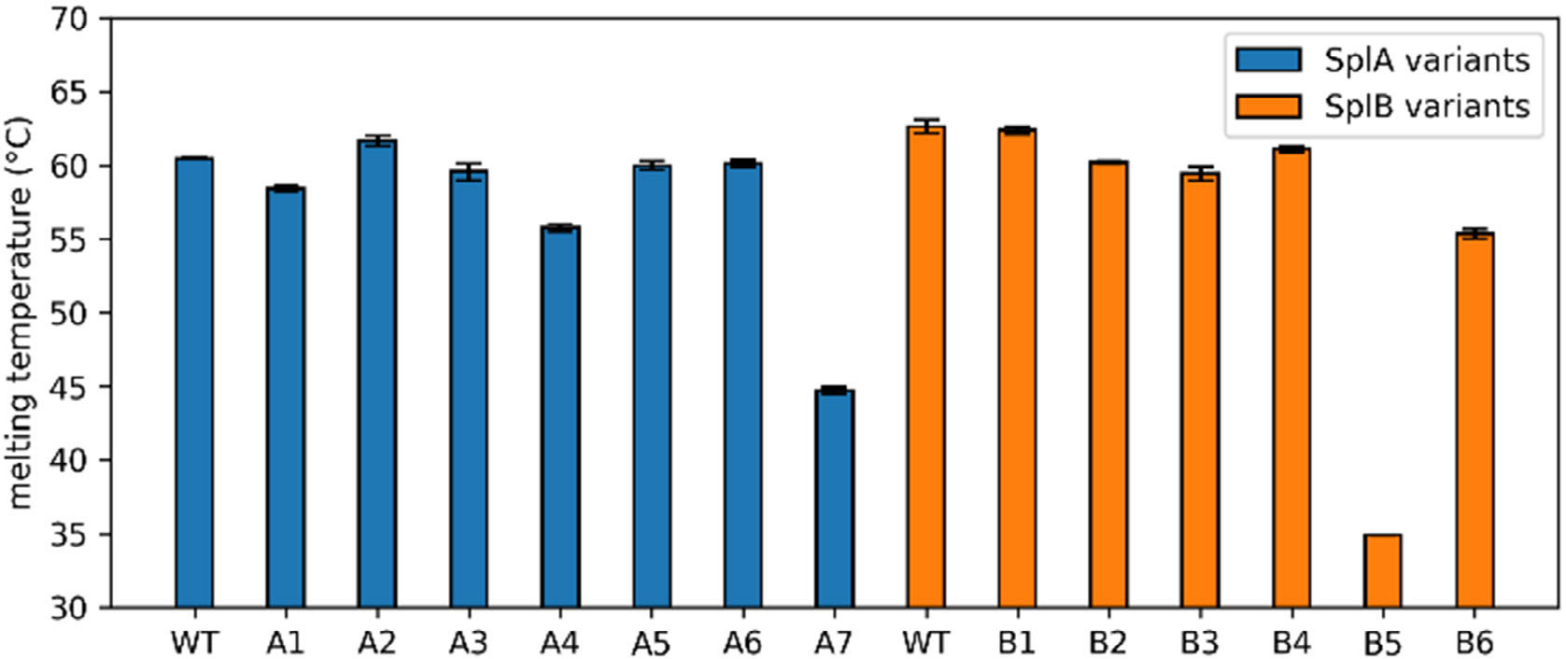
Thermal stability characterization of SplA and SplB allelic variants. The melting temperatures were determined in phosphate buffered saline (PBS, pH 7.4) by nano‐differential scanning fluorimetry. A protein concentration of 1–2 mg mL^−1^ was used for the protein solutions of Spl WTs and allelic variants in the measurements. The experiment was repeated in three independent biological replicates (shown are the mean ± SD).

The SplA WT and SplB WT proteins have a generally high thermal stability^[^ ^22^ ^,^ ^23^ ^]^ with melting temperatures (T_m_) of 60–68 °C, depending on the pH (Figure S1, Supporting Information). Most other Spl allelic variants have similar melting temperatures, with a few exceptions (**Figure** **2**). For example, A7 and B5 showed drastically reduced thermal stability, with their melting temperatures being around 15 and 30 °C lower than the respective Spl WT. Both variants contain a mutation in the GN**S**GSP motif with an exchange of a serine to proline at the homologous position (GN**S**GSP to GN**S**GPP, A7: S156P, B5: S159P). In case of A7, the variant contains eight additional mutations compared to the SplA WT. For B5, the exchange in the motif is the only difference compared to the SplB WT sequence.

### 2.3 Spl Activity and Substrate Specificity

The Spl enzymatic activity was monitored by the proteolytic cleavage of different fluorogenic peptide substrates (Table S3, Supporting Information) that were designed based on the reported cleavage motifs for SplA and SplB (**Table** **1**). Proteolytic cleavage of the scissile bond between the peptide and the fluorophore AMC increases the fluorescence signal, which can be quantified.

**Table 1:** Consensus sequences recognized and cleaved by SplA, SplB, and SplE, determined with cellular libraries of peptide substrates (CLiPS).^[^ ^18^ ^,^ ^25^ ^,^ ^32^ ^]^ P4‐P1′ nomenclature after Schechter and Berger.^[^ ^33^ ^]^

	Consensus sequence				
	P4	P3	P2	P1	P1′
SplA	–	W/Y	L	Y	T/S
SplB	W/V	E	L/I	Q/D	–
SplE	V/L	Y/F/W	L	H/Q	S/G

The activity of SplA and SplB WTs and their allelic variants was determined under various buffer conditions. For the Spl allelic variants that showed activity towards the Spl‐specific substrates, no shift in the pH optimum or preferred buffer conditions were observed compared to the respective WT (data not shown). The SplA variants showed the highest activity in phosphate buffered saline (PBS, pH 7.4) and the SplB variants in 50 mM TRIS buffer (pH 8.2). In the following experiments, Spl activity was therefore monitored under their determined respective optimal buffer conditions.

**Figure 3:**
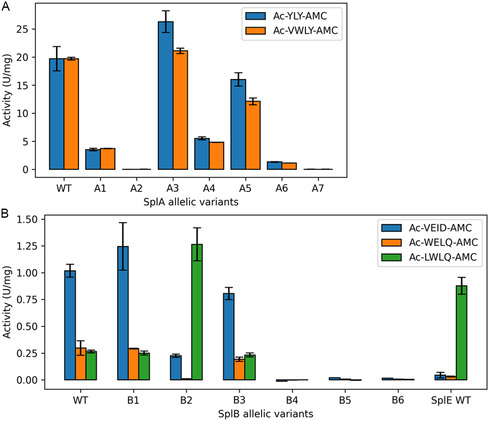
Spl activity and substrate specificity. Specific activity in Units (nmol/min) per mg of A) SplA WT and its allelic variants in PBS (pH 7.4) or B) SplE WT and SplB WT and its allelic variants in 50 mM TRIS buffer (pH 8.2) towards different Ac‐X‐AMC substrates (X = Spl‐specific peptide) at 37 °C. The variant B2 exerts a SplE‐like activity with a decrease in activity towards Ac‐VEID‐AMC, a near complete loss of activity towards Ac‐WELQ‐AMC and a strong increase towards Ac‐LWLQ‐AMC compared to the SplB WT. The experiments were repeated in three independent biological replicates (shown are the mean ± SD).

For SplA, the activity of the variants A3 and A5 was similar to the WT with a slight increase or decrease in activity, respectively (**Figure** **3**A). The variants A1, A4, and A6 showed a drastic decrease in activity compared to the WT, and A2 and A7 did not show any activity towards the SplA‐specific substrates Ac‐YLY‐AMC and Ac‐VWLY‐AMC. The activity towards those two peptide substrates was usually very similar, only variants A3 and A5 showed slightly higher activity towards Ac‐YLY‐AMC compared to Ac‐VWLY‐AMC.

For SplB, the variants B4, B5 and B6 did not cleave any of the three SplB‐specific peptide substrates (Figure 3B). SplB WT, B1 and B3 had very similar activity and specificity, with similar activity towards Ac‐WELQ‐AMC and Ac‐LWLQ‐AMC and higher activity towards Ac‐VEID‐AMC. In contrast, the variant B2 had almost completely lost activity towards Ac‐WELQ‐AMC, while the activity towards Ac‐LWLQ‐AMC showed a strong increase. This represents a significant change in substrate specificity for B2 compared to the SplB WT, such that in terms of activity and specificity, B2 more closely resembles SplE than the other highly prevalent SplB variants (Figure 3B). Since the P1 and P2 residues of the two substrates Ac‐WELQ‐AMC and Ac‐LWLQ‐AMC are identical, the change in specificity for B2 can be localized to the P3 and/or P4 position of the substrates.

### 2.4 Michaelis–Menten Kinetics of Spl Allelic Variants

To quantitatively compare the different allelic Spl variants, we next analyzed the Michaelis–Menten (MM) kinetics. The MM parameters of the SplA allelic variants that showed activity towards the SplA‐specific substrates were determined (**Table** **2**; Figure S2 & S3, Supporting Information). For each SplA variant, the turnover number (k_cat_) for Ac‐VWLY‐AMC was lower than for Ac‐YLY‐AMC. However, the K_M_ was also considerably lower for Ac‐VWLY‐AMC than for Ac‐YLY‐AMC, resulting in a higher affinity of all SplA variants for the Ac‐VWLY‐AMC peptide. Overall, these SplA variants had higher catalytic efficiency (k_cat_/K_M_) for the Ac‐VWLY‐AMC than the Ac‐YLY‐AMC substrate, i.e., lower turnover number is compensated by the higher affinity towards the Ac‐VWLY‐AMC peptide. As also shown in Figure 3, only variant A3 had a slightly higher activity than the SplA WT towards both substrates, while the other variants were less active, with the variant A6 even showing a reduction of ≈95% compared to the WT.

**Table 2:** Michaelis–Menten parameters of SplA allelic variants using the Ac‐YLY‐AMC and Ac‐VWLY‐AMC substrates. The experiment was repeated in two independent biological replicates, in three technical replicates (shown are the mean ± SD).

SplA variant	Ac‐YLY‐AMC			Ac‐VWLY‐AMC		
	k_*cat*_ [ s^−1^]	K_M_ [ μM]	k_*cat*_/K_M_ [ M^−1^ s^−1^]	k_*cat*_ [ s^−1^]	K_M_ [ μM]	k_*cat*_/K_M_ [ M^−1^ s^−1^]
WT	0.0295 (±0.0010)	49 (±7)	601 (±61)	0.0129 (±0.0003)	11 (±2)	1165 (±130)
A1	0.0054 (±0.0004)	54 (±9)	102 (±11)	0.0038 (±0.0002)	26 (±3)	144 (±7)
A3	0.0312 (±0.0006)	39 (±4)	801 (±59)	0.0135 (±0.0006)	11 (±0.7)	1227 (±26)
A4	0.0099 (±0.0010)	65 (±7)	151 (±0.2)	0.0044 (±0.0002)	23 (±4)	193 (±22)
A5	0.0183 (±0.0006)	35 (±2)	519 (±43)	0.0084 (±0.0008)	13 (±0.2)	646 (±48)
A6	0.0033 (±0.0006)	109 (±30)	31 (±3)	0.0009 (±0.0001)	19 (±5)	49 (±6)

The SplB WT has a reported K_M_ of ≈350 µM for Ac‐VEID‐AMC,^[^ ^19^ ^]^ which is much higher than the K_M_ values for the here investigated SplA variants (Table 2). Due to the high K_M_ and low solubility of the SplB‐specific substrates used, a high organic solvent concentration of over 50% DMSO would be required for the assay. The AMC substrates were therefore not applicable for determining MM parameters for the SplB variants.

### 2.5 Proteolytic Activity of Spl Allelic Variants towards Spl Target Proteins

Next, we turned from small synthetic peptides to large protein substrates and studied the cleavage of several deubiquitinases (DUBs) or ubiquitin‐like specific proteases (ULPs) which we have previously identified as targets of the SplA and SplB WTs.^[^ ^12^ ^]^

**Figure 4:**
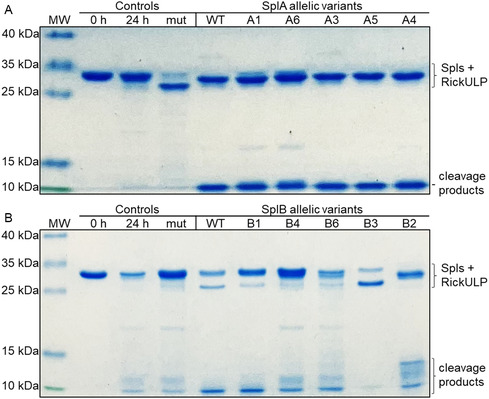
SDS‐PAGE analysis of RickULP cleavage by Spl allelic variants. RickULP was incubated in a 2:1 ratio with A) SplA variants in PBS and B) SplB allelic variants in 100 mM TRIS pH 8.2. All incubations were at 37 °C for 24 h. Controls: target protein before (0 h) and after incubation in absence (24 h) or presence (mut) of inactive Spl mutants (with the catalytic serine exchanged to alanine: SplA_S154A, SplB_S157A). The Spls and the uncleaved RickULP substrate were of similar molecular weight (calculated mass: Spls = 25 kDa, RickULP = 27 kDa). MW = molecular weight marker, contrast and brightness were adjusted for visual clarity.

For initial screening, the target proteins were incubated with each Spl allelic variant at 37 °C for 24 h and analyzed by SDS‐PAGE. Cleavage of the target proteins could be observed by either the disappearance of the target protein band or by the appearance of peptide bands at ≈10 kDa, comprised of smaller cleavage products. In some cases, additional cleavage products of higher molecular weight were observed (**Figures** **4** & S4, Supporting Information). In some instances, bands of possible cleavage products are low in intensity (due to low overall proteolytic activity of some Spl variants) and some target proteins are also prone to partial unspecific degradation under the chosen reaction conditions. In most cases, however, a clear discrimination between no cleavage and Spl‐specific cleavage was possible. The few unclear cases were marked as “possible cleavage” (**Table** **3**). For the allelic variants which did not show any activity towards the fluorogenic peptide substrates (A2, A7 and B4‐B6), no cleavage of the target proteins was observed either. Only for B4 and B6, possible cleavage of the two target proteins RickCE and RickULP was observed suggesting that B4 and B6 evolved to recognize other substrates resembling RickCE and/or RickULP in sequence and/or structure (Figures 4 & S4, Supporting Information).

**Table 3:** Cleavage of DUBs/ULPs by SplA and SplB allelic variants. x = cleavage, (x) = possible cleavage (unclear due to low intensity of possible cleavage products and unspecific degradation of target proteins), ‐ = no cleavage. Variants A2, A7, and B5 did not show any cleavage and are excluded from the table. The experiment was repeated in three independent biological replicates.

Target proteins	SplA variants						SplB variants					
	WT	A1	A3	A4	A5	A6	WT	B1	B2	B3	B4	B6
ElaD	x	–	x	–	x	–	–	–	–	(x)	–	–
Lpg1148	x	(x)	x	(x)	x	–	x	x	x	x	–	–
Lpg1621	x	–	x	–	x	–	x	–	–	x	–	–
RickCE	x	(x)	x	(x)	x	–	x	x	x	x	–	(x)
RickULP	x	x	x	x	x	x	x	x	x	–	(x)	(x)
SnCE1	–	–	–	–	–	–	–	–	–	x	–	–
SseL	x	x	–	x	x	–	x	x	–	x	–	–

**Figure 5:**
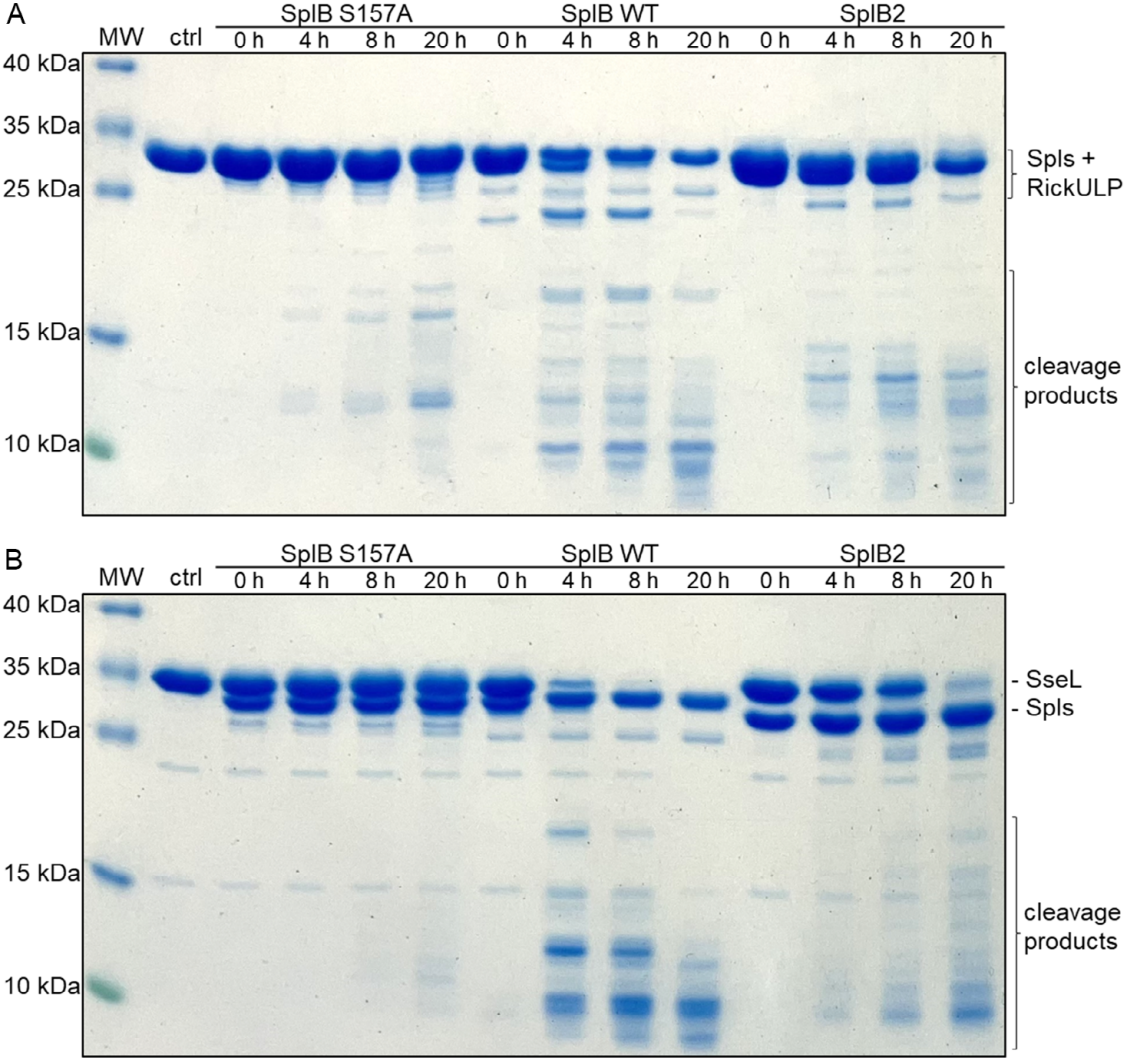
The SplB WT and the SplB variant B2 generate different cleavage patterns when targeting the same protein substrates. SDS‐PAGE analysis of A) RickULP and B) SseL cleavage by SplB variants. The target proteins were incubated with the inactive SplB mutant S157A (with the catalytic serine exchanged for alanine), the SplB WT, or the allelic variant B2. All incubations were performed at 37 °C in 100 mM TRIS pH 8.2. The cleavage efficiency and cleavage patterns differed between SplB WT and variant B2. MW = molecular weight marker, ctrl = target protein prior to incubation; contrast and brightness were adjusted for visual clarity.

This screening revealed that the Spl allelic variants have distinctive profiles of which target proteins are cleaved, signifying that the differences in the Spl sequences changed their substrate recognition or specificity. When cleaving the same substrate as the Spl WT, the Spl variants mostly generated substrate fragments of the same size, but in some instances, different fragments or cleavage patterns were observed. As shown above (Figure 3), the SplB variant B2 differed from the WT and the B1 and B3 variants in its preference for synthetic peptide substrates. In line with this, B2 also generated unique cleavage patterns in RickULP and SseL. The cleavage of these two targets by the SplB WT and B2 was then kinetically investigated (**Figure** **5**). Again, clear differences in the cleavage pattern between SplB WT and B2 could be observed. RickULP showed some unspecific degradation when incubated with a proteolytically inactive SplB mutant (with the catalytic serine S157 exchanged to alanine), but the resulting cleavage patterns when incubated with SplB WT or B2 are still clearly distinguishable (Figure 5A).

### 2.6 Spl‐Specific Cleavage Site Identification in RickULP

To further characterize the cleavage motifs of the SplB WT and B2, we used site‐directed mutagenesis (SDM) of the target protein RickULP. Based on the reduced activity of the SplB variant B2 towards the Ac‐VEID‐AMC substrate with aspartate at the P1 position and the apparent size of the cleavage products of RickULP when subjected to cleavage by SplB (Figure 5A), three aspartate residues within the RickULP sequence were identified as potential SplB cleavage sites (Figure S5, Supporting Information). Since two of these residues are located in immediate vicinity, we generated a D87A+D88A double mutant and a D98A single mutant to study these potential cleavage sites. Both RickULP mutants with changes in the potential SplB‐specific cleavage sites were still cleaved by the SplA WT, generating the same cleavage pattern as for the RickULP WT (Figure S6, Supporting Information). This indicates that the overall protein folding was not significantly impacted by the introduced mutations. Thus, the Spl‐specific cleavage sites should still be accessible to the enzymes.

**Figure 6:**
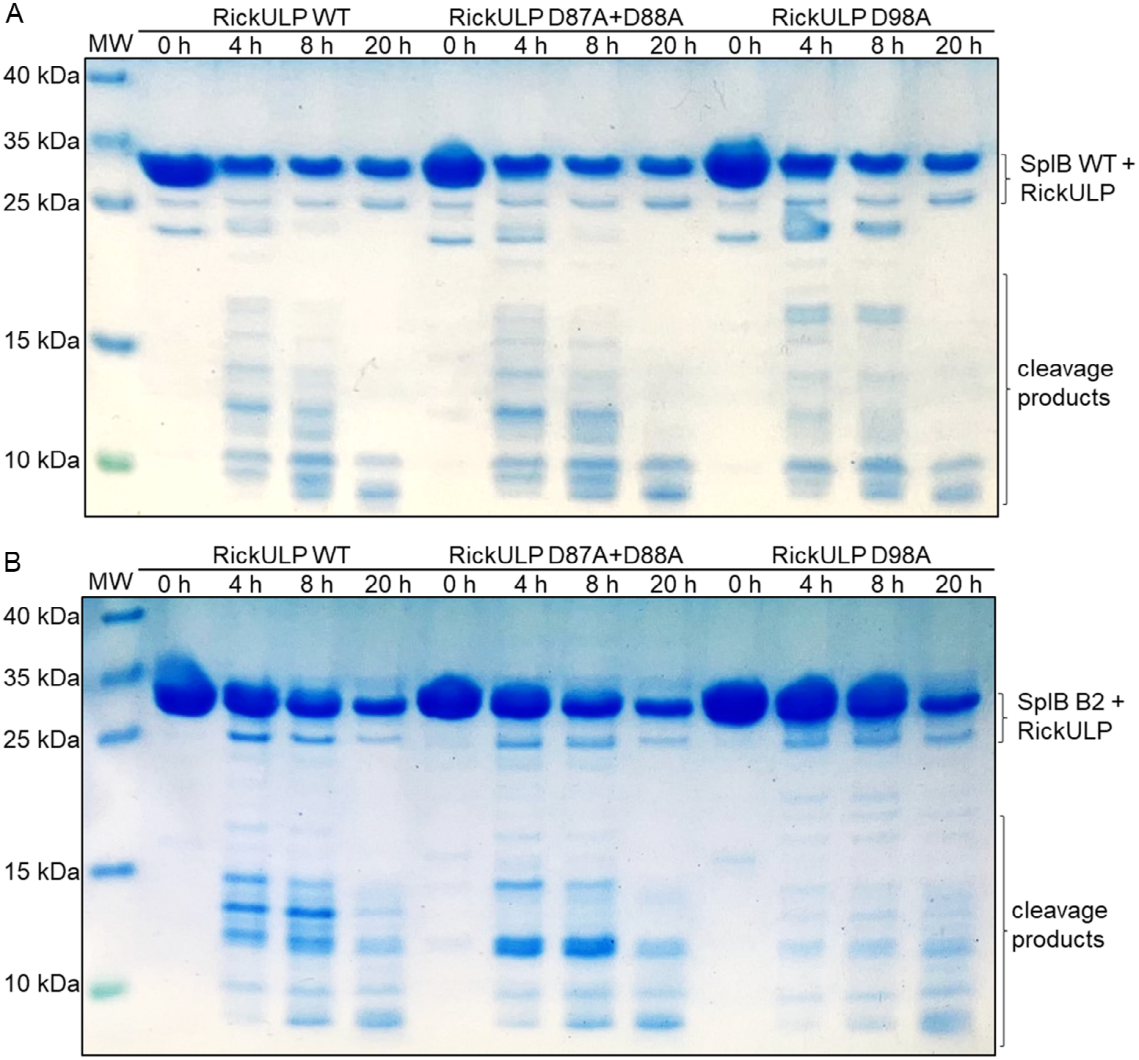
Spl‐specific cleavage site identification in RickULP. SDS‐PAGE analysis of RickULP WT, D87A+D88A double mutant and D98A single mutant cleavage by A) SplB WT and variant B) B2. The RickULP variants were incubated with the Spls at 37 °C in 100 mM TRIS pH 8.2. Different cleavage patterns are observed for the SplB WT and variant B2. MW = molecular weight marker; contrast and brightness were adjusted for visual clarity.

When incubated with the SplB WT, the cleavage patterns for the RickULP WT and D87A+D88A double mutant looked identical, excluding these positions as cleavage sites of SplB WT (**Figure** **6**A). In contrast, cleavage of the RickULP D98A mutant resulted in larger, less degraded fragments after 4 h and 8 h. After 20 h, however, the target protein was still fully degraded like the RickULP WT. This implies, that D98 is a prominent cleavage site of SplB WT in RickULP. Disabling this cleavage site changes the fragment pattern generated during the degradation of RickULP, while the complete digestion ultimately results in fragments of similar size (≈10 kDa).

The cleavage of RickULP by the SplB variant B2 differed from that by the SplB WT in several respects. First, cleavage was much slower; second, the cleavage pattern of the RickULP WT differed from that generated by the SplB WT; and third, both RickULP mutations changed the cleavage patterns generated by B2 (Figure 6B). In particular, a prevalent ≈13 kDa cleavage fragment of the RickULP WT was not observed in the mutants. This shows that, unlike SplB WT, B2 recognizes and cleaves at both examined positions.

The amino acid residue D98 is located on the surface of RickULP, making it easily accessible for cleavage by SplB. In contrast, D87 and D88 appear to be occluded by a large loop region of RickULP (Figure S5, Supporting Information). These two residues are probably only exposed to SplB after prior cleavage at a different position. This could explain why digestion of RickULP by B2 at position D87/D88 is much slower and less complete when position D98 is mutated. The different cleavage motif of B2 compared to the SplB WT is likely due to the preferred/more efficient cleavage of motifs with glutamine residues at the P1 position instead of aspartate, as shown in the peptide assay (Figure 3).

## 3 Discussion

Allelic variation of pathogens, especially in their virulence factors, is one of the key mechanisms for adapting to different hosts and their defense mechanisms. In this study, we characterized several allelic variants of the *S. aureus* serine protease‐like proteins SplA and SplB with regard to their stability, activity, substrate specificity, and their cleavage motifs, where we found some striking differences. This highlights the importance of considering allelic variation of pathogens and their virulence factors when studying host–pathogen interaction.

The Spl allelic variants originate from clinical *S. aureus* strains. The composition of the *spl* operon differs between the strains which may affect the levels of transcription and regulation of *splA‐F*.^[^ ^10^ ^]^ In our biochemical characterization of the Spl allelic variants, we focused on differences in the mature Spl protein sequences. Variations in the signal peptide or silent mutations (nucleotide exchanges that result in the same encoded amino acid) could also influence the expression and secretion of Spls in their natural context. Similarly, interplay with other secreted virulence factors cannot be ruled out. Still, the identification of Spl cleavage motifs and respective changes in specificity among Spl allelic variants offers deep insight into the spectrum of potential targets and the role of the Spls in *S. aureus* interaction with its host.

Most of the investigated Spl variants, especially the most frequent ones, originate from methicillin‐resistant *S. aureus* (MRSA) strains (Table S4, Supporting Information). Due to their clinical and public health implications, MRSA strains are generally investigated more frequently than other *S. aureus* strains.^[^ ^24^ ^]^ This results in an abundance of sequence data for MRSA strains and the frequency of the Spl variants in the RefSeq database likely does not reflect the actual frequency or natural occurrence of different Spl variants.

Several fluorogenic peptide substrates for the SplA WT and SplB WT were designed for this study based on their respective reported cleavage motifs (Table 1).^[^ ^18^ ^,^ ^25^ ^]^ SplA WT and its allelic variants did not show any activity towards the SplB‐specific substrates and vice versa. The enzyme kinetics of several SplA allelic variants were determined, allowing a more detailed comparison of their activity and the observed differences among the variants. The SplA WT exhibited a catalytic efficiency (k_cat_/K_M_) of 1165 ± 130 M^−1^ s^−1^ for the Ac‐VWLY‐AC substrate (Table 2). The only other reported value for the same substrate under almost the same conditions is 530 ± 136 M^−1^ s^−1^.^[^ ^21^ ^]^ Besides the relatively high SD, this discrepancy can probably be attributed to slight differences in buffer conditions and temperature. As described above, the high K_M_ of SplB using our short peptide substrates would result in organic solvent concentrations exceeding 50% DMSO or acetonitrile, making it not feasible to determine SplB's kinetics with these substrates. Similar limitations for SplB were also described by Pustelny et al.^[^ ^19^ ^]^ In their study, conventional parameters of Michaelis–Menten kinetics (k_cat_ and K_M_) were measurable only for the substrate Ac‐VEID‐AMC, which required excessive amounts of substrate due to the high K_M_ of 352 ± 25 µM (k_cat_/K_M_ = 30 ± 2 M^−1^ s^−1^). For Ac‐WELQ‐AMC, the group performed inverse MM kinetics (varying enzyme concentrations rather than substrate concentrations), yielding only the catalytic efficiency (k_cat_/K_M_) of the SplB WT to be 955 ± 18 M^1^ s^1^.^[^ ^19^ ^]^ Another group reported 2690 ±  525 M^−1^ s^−1^ as the value for the catalytic efficiency for the SplB WT towards Ac‐WELQ‐AMC, albeit with higher SD.^[^ ^21^ ^]^

Under their respective optimal reaction conditions, the SplA WT had an activity of 20 units ( nmol min^−1^) per mg protein, which was around 20‐fold higher than for the SplB WT with 1 unit mg^−1^. While the activity of the SplA WT is comparable to the reported activity of commercial enterokinase (Sigma Aldrich, Catalog Number E4906: ≥20 nmol of trypsin from trypsinogen per min per mg protein), the proteolytic activity of SplA and especially SplB is generally very low. This could be of advantage for *S. aureus*, since pathogens can benefit from low proteolytic activity of their secreted virulence factors. A lower level of proteolytic activity may be less likely to trigger a strong immune response and may also be less likely to degrade essential immune factors, such as antimicrobial peptides, balancing the interaction of the pathogen with its host.^[^ ^26^ ^,^ ^27^ ^]^ It is also possible that the Spls are dual‐function “moonlighting” proteases, exhibiting both proteolytic and non‐proteolytic activities. This phenomenon has been observed for several proteases, with examples in cellular quality control, metabolism and immunity.^[^ ^28^ ^,^ ^29^ ^]^

The Spl variants which did not show any activity towards the respective SplA WT‐ or SplB WT‐specific short peptide substrates (Figure 3) either contain a variant site in the highly conserved GNSGSP motif that contains the catalytic serine (A2: G152C, A7: S156P, B5: S159P, B6: G156R) or have a residue of the catalytic triad mutated (B4: H39N). These variants are therefore likely proteolytically inactive due to disruption of the catalytic triad. Variants A7 and B5 also showed drastically reduced thermal stability (Figure 3), further highlighting the importance of the conserved GNSGSP motif for the correct folding and functioning of the Spls. Only variant A2 has a variant site in a loop region that builds the S1 substrate specificity pocket (G152C). This modification likely impacts the binding of SplA WT‐specific substrates and the loss in activity could therefore indicate a change in specificity (in the S1 pocket) instead of a general inactivation of its proteolytic activity. Although the side chain of C152 does not protrude into the S1 pocket of variant A2 (Figure 1), loop structures are rather flexible and the exchange of glycine to cysteine likely impacts its freedom of movement.

When tested against several peptide substrates, the SplB allelic variant B2 exhibited a significant change in substrate preference compared to the SplB WT, such that B2 activity and specificity more closely resemble those of the SplE WT (USA300) than those of the other highly prevalent SplB variants. Compared to the SplB WT, the variant B2 contains a total of 24 point mutations distributed throughout the entire protein, resulting in a pairwise sequence identity of 88.2%. Although B2's activity and substrate specificity more closely resemble those of the SplE WT than the SplB WT, its sequence is still as distant from the SplE WT as is that of the SplB WT, with 52.2 and 52.0% sequence identity with SplE, respectively. While the overall protein fold of the B2 variant is nearly identical to the SplB WT, several mutations are located in a loop structure that builds part of the S1 pocket (Figure 1). The electrostatic surface potential of B2 also differs from the SplB WT, with a more neutral or slightly positive charge of the active site cleft and S1 pocket, which more closely resembles the SplE WT (Figure S7, Supporting Information). These changes in and around the active site likely impact the substrate recognition of variant B2 and may explain its SplE WT‐like activity. The activity of the B2 variant towards Ac‐LWLQ‐AMC was strongly increased compared to the SplB WT, while no activity towards Ac‐WELQ‐AMC was detectable anymore. The change in specificity for B2 thereby can be localized to the P3 and/or P4 position of the substrates. Molecular modeling and substrate dockings were performed (data not shown), but the specificity beyond the P1 and P2 positions could not be satisfactorily explained by modeling, suggesting that an induced fit mechanism might be involved. The same limitations of molecular modeling approaches were previously described for SplA and SplB regarding their specificity beyond the P1 and P2 position.^[^ ^18^ ^,^ ^25^ ^]^

In this study, we demonstrate that Spl allelic variants, while targeting the same protein substrates, may generate different cleavage patterns. This was especially apparent for the cleavage of RickULP and SseL by the SplB WT and variant B2 (Figure 5). B2 has two previously unknown cleavage sites in RickULP, while the SplB WT only targets one of them (Figure 6). This clearly shows that SplB allelic variants may differ in their cleavage motifs. Some differences in the observed cleavage patterns may be explained by kinetic differences rather than absolute differences in selectivity. However, multiple cleavage fragments were only observed when incubated with a specific Spl variant instead of appearing after longer incubation times or at lower intensity. Additionally, the cleavage patterns of RickULP by SplB after the exchange of SplB‐specific putative cleavage sites were significantly altered (Figure 5), signifying a clear specificity or at least strong preference towards these cleavage sites within RickULP.

The number of cleavage fragments and the complete digestion into small ( ≤10 kDa) fragments over extended incubation times suggest that RickULP and SseL contain several additional SplB‐specific cleavage sites, besides those that were previously reported^[^ ^12^ ^]^ or identified in this study. However, it proved very difficult to demonstrate additional putative SplB cleavage sites in these target proteins by site‐directed mutagenesis (SDM). Most of the introduced mutations (mostly exchange of the putative P1 reside to alanine) resulted in significant alterations in the overall protein fold or structural integrity of the target proteins. Many variants that were generated by SDM could not be expressed in a soluble form and others showed dramatic decreases in thermal or overall protein stability. The variants which behaved like the native target proteins were subjected to cleavage by the Spls, but besides the examples shown in this study and the already reported confirmed cleavage sites,^[^ ^12^ ^]^ the resulting cleavage patterns were conserved in SDS‐PAGE analysis. This could of course be due to the different detection limits of the methods used (MS vs. Coomassie staining). Cleavage products that are detectable by MS may not be visible on a gel, and, conversely, there is a risk of false positives in MS. It is also possible that the determined cleavage sites are correct, but the exchange of the P1 residue to alanine does not prevent cleavage. The published SplA and SplB cleavage motifs were determined using peptide libraries instead of naturally folded protein substrates,^[^ ^18^ ^,^ ^25^ ^]^ and there are known examples of proteases recognizing structure motifs rather than a fixed P1 amino acid sequence. An example for this is the HtrA serine peptidase 1 (HTRA1), which recognizes the overall helical structure of substrates like annexin A1.^[^ ^30^ ^]^ Structural analysis of the identified Spl cleavage sites in actual target proteins^[^ ^10^ ^,^ ^12^ ^]^ revealed that the enzymes attack either unstructured or helical regions to a fairly equal extent, suggesting a preference towards specific structural elements instead of sequence motifs. To further investigate this, we performed a saturation mutagenesis (mutant library created with the degenerate “NDT” codon)^[^ ^31^ ^]^ of the P1 residue in one of the two previously identified SplA cleavage sites that is located in a helical region of RickULP.^[^ ^12^ ^]^ After incubation with SplA, no cleavage could be observed at this position for any residue besides the native tyrosine (data not shown), which suggests a fixed P1 preference (at least for SplA). This approach was therefore not applied further for the other identified cleavage sites.

## 4 Conclusion

Our biochemical characterization of selected SplA and SplB allelic variants shows that the *spl* operon of *S. aureus* is a cradle of evolutionary change, which affects the function and substrate preference of the encoded proteases. This approach opens an exciting new avenue for elucidating the development of sequence‐structure‐function relationships of naturally occurring enzymes. Our study reveals that allelic variation adds an extra layer of complexity to the functioning of virulence factors in *S. aureus* colonization and infection. It highlights the importance of considering allelic variation in the study of host‐pathogen interactions.

## 5 Experimental Section

5.1

5.1.1

##### Bacterial Strains and Plasmids

*Escherichia coli* strains TOP10 and BL21(DE3) were used for genetic manipulations and protein expression, respectively. The spl genes of *S. aureus* strain USA300 with an N‐terminal SUMO‐tag and C‐terminal Twin‐Strep‐tag were ordered in a pET‐28a(+) vector (BioCat GmbH, Heidelberg, Germany). The propeptide sequences were retained, while the signal peptide sequences were excluded in the design of the synthetic genes. The bacterial DUB and ULP genes were ordered from BioCat in a pET‐45b(+) vector. The sequences were codon‐optimized for expression in *E. coli*. The SENP1 gene in a pOPINS vector was kindly provided by Kay Hofmann (University of Cologne, Institute for Genetics). Sequences of the constructs are provided in the Supporting Information.

##### Recombinant Production of Proteins

SENP1, bacterial DUBs, and Spl constructs were produced in *E. coli* BL21(DE3). The cells were grown in TB‐AIM (TB, 25 mM Na_2_HPO_4_, 25 mM KH_2_PO_4_, 50 mM NH_4_Cl, 5 mM Na_2_SO_4_, 2 mM MgSO_4_, 0.5% (w/v) glycerol, 0.2% (w/v) lactose, and 0.05% (w/v) glucose) at 37 °C until an OD_600_ of 0.8 was reached, at which point the temperature was reduced to 20 °C overnight. For SUMO‐tag removal, the cell cultures of Spls and SENP1 were combined in a 2:1 ratio (v/v), centrifuged at 10,000 g and 4 °C for 30 min, resuspended in 100 mM TRIS (pH 8) and 150 mM NaCl, lysed by sonication, and the lysate was incubated for 1 h at 4 °C.

##### Protein Purification

Recombinant Spls and target proteins were purified from the lysate by gravity‐flow column chromatography. For Spl proteins, Strep‐Tactin XT resin (IBA Lifesciences) was used. The column was equilibrated and washed with 100 mM TRIS buffer, 150 mM NaCl, pH 8. The elution buffer contained additional 50 mM biotin. The target proteins (DUBs/ULPs) were purified by immobilized‐metal affinity chromatography (IMAC) using ROTI Garose‐His/Ni Beads (Carl Roth GmbH). The column was equilibrated with 20 mM TRIS buffer, 300 mM NaCl, and 10 mM imidazole, pH 7, and washed with 20 mM TRIS buffer, 500 mM NaCl, and 20 mM imidazole, pH 7. The protein was eluted using 20 mM TRIS buffer, 300 mM NaCl, and 250 mM imidazole, pH 7. Eluted protein solutions were buffer exchanged to phosphate buffered saline (PBS, pH 7.4) using PD‐10 columns (Sephadex G‐25 resin, VWR), followed by a concentration using spin concentrators (10 kDa MWCO). The purity of the proteins was validated by SDS‐PAGE analysis, and a Bradford assay was used to determine the protein concentration (ROTINanoquant, Carl Roth GmbH).

##### Thermal Stability Characterization

Thermal stability of Spl proteins was determined in PBS or 50 mM sodium phosphate buffer (pH 6–8) by nano‐differential scanning fluorimetry (nanoDSF) using the Prometheus NT.48 device (NanoTemper Technologies GmbH, Munich, Germany). It uses intrinsic protein fluorescence to monitor structural changes (folding/unfolding), while applying a defined temperature profile. The Spl samples were set to a concentration of 1–2 mg mL^−1^ and heated from 20–95 °C at 1 °C min^−1^.

##### Fluorescence‐Based Activity Assays

A substrate‐specific activity assay was conducted using synthetic peptide substrates that are covalently linked to a 7‐amino‐4‐methylcoumarin (AMC) and contain the respective cleavage sites for SplA^[^ ^18^ ^]^ or SplB^[^ ^25^ ^]^ (Tables 1 & S2, Supporting Information). Upon proteolytic cleavage of the scissile bond between AMC and the attached peptide, AMC is released and fluoresces, which can be quantified in a fluorescence reader. In the assay, 90 µl of 25 µM substrate was incubated in a microtiter plate (96 well polystyrene microplate, black; Greiner Bio‐One, REF: 655,077) with 10 µl of 2.5 µM Spls in PBS (pH 7.4) or 100 mM TRIS (pH 8.2) at 37 °C for up to 1 h. The fluorescent AMC signal was continuously measured (usual interval of 1 min) on a TECAN Infinite M200 plate reader (Tecan Group Ltd., Maennedorf, Switzerland; excitation: 360 nm, emission: 460 nm). A standard curve of the free AMC product was used to convert the change in fluorescence signal into the amount of released AMC. For kinetic measurements, AMC substrate concentrations of 6.25, 12.5, 25, 50, 100, and 150 mM were used. The initial velocities were calculated using a polynomial fit (numpy.polyfit, first degree).

##### Proteolytic Activity Assays

If not stated otherwise, the target proteins were incubated with Spls in a 2:1 molar ratio at a concentration of 1 mg mL^−1^ in PBS, pH 7.4 (SplA), or 100 mM TRIS, pH 8.2 (SplB), at 37 °C for up to 24 h. The reaction was terminated by adding denaturing SDS sample buffer, and the samples were then analyzed by SDS‐PAGE.

## Conflict of Interest

The authors declare no conflict of interest.

## Supporting information

Supplementary Material

## Data Availability

The data that support the findings of this study are available in the supplementary material of this article.
