# Supplementary material for: Staphylococcal SplA and SplB Serine Protease Allelic Variants Exhibit Different Substrate Specificities
Source: Chembiochem. 2025 Dec 22;27(1):e202500816. doi: 10.1002/cbic.202500816 (PMC12783888; doi:10.1002/cbic.202500816)
Supplement: Supplementary file 1 — Supplementary Material [file CBIC-27-e202500816-s001.pdf]

Table of Contents

Selection of Spl allelic variants 2

Sequence homology of Spl WTs 3

Amino acid sequences of protein constructs 4

Synthetic peptide substrates used in activity assays 6

Thermal stability of SplA and SplB WTs 6

Michaelis-Menten kinetics of SplA allelic variants 7

Initial substrate screening for SplA and SplB WTs 9

Protein structure of Spl target protein RickULP 10

Cleavage of RickULP mutants by SplA WT 10

Protein structure and electrostatic surface potential of Spls 11

S. aureus lineages of investigated Spl variants 12

Contributor Roles 12

Selection of Spl allelic variants

The allelic variants of SplA and SplB studied in this work are listed in Table S1. They were mostly selected based on their frequency (amount of *spl* operons variants encoding for the respective protein sequence) or due to variations in or near the catalytic center.

Table S1 SplA and SplB allelic variants used in this study. Name/abbreviation of each variant, its frequency (amount of *spl* operons encoding for the respective Spl variant) and reason for selection. * = Spl variant was only produced in an insoluble form, ** = based on literature[22].

Spl variant	Mutations (compared to WT)	RefSeq accession number	Frequency (out of 9,230)	Reason for selection
SplA WT	-	GCF_902723625.1	3,216	SplA of *S. aureus* USA300 (termed WT in this study)
A1	E13A, V22A, S52A, H55Y, D105E, V109A, M143I	GCF_910590185.1	3,312	prevalent allelic variant
A2	E13A, V22A, S52A, H55Y, D105E, V109A, M143I, G152C	GCF_903932605.1	228	prevalent allelic variant
A3	V22A	GCF_910590205.1	190	prevalent allelic variant
A4	E13A, A20T, V22A, D45N, V109A, S139N, H163N, F182Y	GCF_014267285.1	165	prevalent allelic variant
A5	E13A, V22A, D45N, S52A, V109A, S139N	GCF_019458405.1	127	prevalent allelic variant
A6	D78E	GCF_003422405.1	3	mutation in catalytic triad
A7	E13A, A20T, V22A, D45N, V109A, S139N, S156P, H163N, F182Y	GCF_900125645.1	1	mutation in GNSGSP motif
A8*	E13A, V22A, S56L, D105E, V109A, D146G, G155E	GCF_015732455.1	251	mutation in GNSGSP motif
A9*	E13A, V22A, S52A, H55Y, D105E, V109A, M143I	GCF_000576775.1	12	most prevalent truncated allelic variant
SplB WT	-	GCF_902723625.1	3,716	SplB of *S. aureus* USA300 (termed WT in this study)
B1	V7I, K8Q	GCF_910590185.1	2,956	prevalent allelic variant
B2	F21L, K22S, N43Y, S65T, I70T, N71D, Q82H, I88V, A112I, H123E, V130I, Y132H, M139A, V141I, E142N, T152V, S154V, F173Y, V177A, K178N, D180G, D181N, R183K, A185G	GCF_900250735.1	49	mutation with possible impact on activity**
B3	K22N, V29I, G59S, Q82K, R86H, E89K, E114D, Y132H	GCF_009912935.1	3	mutation with possible impact on activity**
B4	H39N	GCF_900083175.1	1	mutation in catalytic triad

B5	S159P	GCF_014061235.1	1	mutation in GNSGSP motif
B6	N11S, A20L, F21V, K22N, N43Y, T51N, S56G, G59A, N71D, K93N, F95Y, E114D, H123Q, Y125H, V130I, Y132H, G158R, N179D	GCF_000162635.1	1	mutation in GNSGSP motif
B7*	V7I, K22N, I50T, G59S, A112D, E114D, Y132H, S154R	GCF_009696775.1	1	mutation with possible impact on activity**

Sequence homology of Spl WTs

Table S2 Sequence homology of SplA-F WT sequences in %. Adapted from Reed *et al.*[9]

	SplA	SplB	SplC	SplD	SplE	SplF
**SplA**	100	47.7	50.0	43.9	43.9	44.8
**SplB**		100	62.9	54.4	56.2	53.9
**SplC**			100	49.8	50.4	51.0
**SplD**				100	67.4	94.6
**SplE**					100	67.8
**SplF**						100

Amino acid sequences of protein constructs

Spl constructs

The Spl constructs contain an N-terminal SUMO-tag which is cleaved off after expression, and a C-terminal TWIN-Strep-tag (marked grey). Variations in the amino acid sequence of SplA and SplB allelic variants compared to their respective WT are marked in bold.

SUMO-tag (SMT3):
MGHHHHHHGSGLVPRGSASMSDSEVNQEAKPEVKPEVKPETHINLKVSDGSSEIFFKIKKTTPLRRLMEAFAKRQ
GKEMDSLRFLYDGIRIQADQTPEDLDMEDNDIIEAHREQIGG

SplA WT (GenBank accession number: WP_399659753.1):
EKNVKEITDATKEPYNSVVAFVGGTGVVVGKNTIVTNKHIAKSNDIFKNRVSAHHSSKGKGGGNYDVKDIVEYPG
KEDLAIVHVHETSTEGLNFNKNVSYTKFADGAKVKDRISVIGYPKGAQTKYKMFESTGTINHISGTFMEFDAYAQ
PGNSGSPVLNSKHELIGILYAGSGKDESEKNFGVYFTPQLKEFIQNNIEKGSWSHPQFEKGGGSGGGSGGSSAWS
HPQFEK

SplA variant A1:
EKNVKEITDATK**A**PYNSVVAF**A**GGTGVVVGKNTIVTNKHIAKSNDIFKNRV**A**AH**Y**SSKGKGGGNYDVKDIVEYPG
KEDLAIVHVHETSTEGLNFNKNVSYTKFA**E**GAK**A**KDRISVIGYPKGAQTKYKMFESTGTINHISGTF**I**EFDAYAQ
PGNSGSPVLNSKHELIGILYAGSGKDESEKNFGVYFTPQLKEFIQNNIEKGSWSHPQFEKGGGSGGGSGGSSAWS
HPQFEK

SplA variant A2:
EKNVKEITDATK**A**PYNSVVAF**A**GGTGVVVGKNTIVTNKHIAKSNDIFKNRV**A**AH**Y**SSKGKGGGNYDVKDIVEYPG
KEDLAIVHVHETSTEGLNFNKNVSYTKFA**E**GAK**A**KDRISVIGYPKGAQTKYKMFESTGTINHISGTF**I**EFDAYAQ
P**C**NSGSPVLNSKHELIGILYAGSGKDESEKNFGVYFTPQLKEFIQNNIEKGSWSHPQFEKGGGSGGGSGGSSAWS
HPQFEK

SplA variant A3:
EKNVKEITDATKEPYNSVVAF**A**GGTGVVVGKNTIVTNKHIAKSNDIFKNRVSAHHSSKGKGGGNYDVKDIVEYPG
KEDLAIVHVHETSTEGLNFNKNVSYTKFADGAKVKDRISVIGYPKGAQTKYKMFESTGTINHISGTFMEFDAYAQ
PGNSGSPVLNSKHELIGILYAGSGKDESEKNFGVYFTPQLKEFIQNNIEKGSWSHPQFEKGGGSGGGSGGSSAWS
HPQFEK

SplA variant A4:
EKNVKEITDATK**A**PYNSVV**T**F**A**GGTGVVVGKNTIVTNKHIAKSN**N**IFKNRVSAHHSSKGKGGGNYDVKDIVEYPG
KEDLAIVHVHETSTEGLNFNKNVSYTKFADGAK**A**KDRISVIGYPKGAQTKYKMFESTGTINHI**N**GTFMEFDAYAQ
PGNSGSPVLNSK**N**ELIGILYAGSGKDESEKN**Y**GVYFTPQLKEFIQNNIEKGSWSHPQFEKGGGSGGGSGGSSAWS
HPQFEK

SplA variant A5:
EKNVKEITDATK**A**PYNSVVAF**A**GGTGVVVGKNTIVTNKHIAKSN**N**IFKNRV**A**AHHSSKGKGGGNYDVKDIVEYPG
KEDLAIVHVHETSTEGLNFNKNVSYTKFADGAK**A**KDRISVIGYPKGAQTKYKMFESTGTINHI**N**GTFMEFDAYAQ
PGNSGSPVLNSKHELIGILYAGSGKDESEKNFGVYFTPQLKEFIQNNIEKGSWSHPQFEKGGGSGGGSGGSSAWS
HPQFEK

SplA variant A6:
EKNVKEITDATKEPYNSVVAFVGGTGVVVGKNTIVTNKHIAKSNDIFKNRVSAHHSSKGKGGGNYDVKDIVEYPG
KE**E**LAIVHVHETSTEGLNFNKNVSYTKFADGAKVKDRISVIGYPKGAQTKYKMFESTGTINHISGTFMEFDAYAQ
PGNSGSPVLNSKHELIGILYAGSGKDESEKNFGVYFTPQLKEFIQNNIEKGSWSHPQFEKGGGSGGGSGGSSAWS
HPQFEK

SplA variant A7:
EKNVKEITDATK**A**PYNSVV**T**F**A**GGTGVVVGKNTIVTNKHIAKSN**N**IFKNRVSAHHSSKGKGGGNYDVKDIVEYPG
KEDLAIVHVHETSTEGLNFNKNVSYTKFADGAK**A**KDRISVIGYPKGAQTKYKMFESTGTINHI**N**GTFMEFDAYAQ
PGNSG**P**PVLNSK**N**ELIGILYAGSGKDESEKN**Y**GVYFTPQLKEFIQNNIEKGSWSHPQFEKGGGSGGGSGGSSAWS
HPQFEK

SplB WT (GenBank accession number: WP_271285242.1):
ENNVTKVKDTNIFPYTGVVAFKSATGFVVGKNTILTNKHVSKNYKVGDRITAHPNSDKGNGGIYSIKKIINYPGK
EDVSVIQVEERAIERGPKGFNFNDNVTPFKYAAGAKAGERIKVIGYPHPYKNKYVLYESTGPVMSVEGSSIVYSA
HTESGNSGSPVLNSNNELVGIHFASDVKNDDNRNAYGVYFTPEIKKFIAENIDKGSWSHPQFEKGGGSGGGSGGS
SAWSHPQFEK

SplB variant B1:
ENNVTK**IQ**DTNIFPYTGVVAFKSATGFVVGKNTILTNKHVSKNYKVGDRITAHPNSDKGNGGIYSIKKIINYPGK
EDVSVIQVEERAIERGPKGFNFNDNVTPFKYAAGAKAGERIKVIGYPHPYKNKYVLYESTGPVMSVEGSSIVYSA
HTESGNSGSPVLNSNNELVGIHFASDVKNDDNRNAYGVYFTPEIKKFIAENIDKGSWSHPQFEKGGGSGGGSGGS
SAWSHPQFEK

SplB variant B2:
ENNVTKVKDTNIFPYTGVVA**LS**SATGFVVGKNTILTNKHVSK**Y**YKVGDRITAHPNSDKGNGGIY**T**IKKI**TD**YPGK
EDVSVI**H**VEERA**V**ERGPKGFNFNDNVTPFKYAAGAK**I**GERIKVIGYP**E**PYKNKY**I**L**H**ESTGPV**A**S**IN**GSSIVYSA
H**V**E**V**GNSGSPVLNSNNELVGIH**Y**ASD**AN**N**GN**N**K**N**G**YGVYFTPEIKKFIAENIDKGSWSHPQFEKGGGSGGGSGGS
SAWSHPQFEK

SplB variant B3:
ENNVTKVKDTNIFPYTGVVAF**N**SATGFV**I**GKNTILTNKHVSKNYKVGDRITAHPNSDK**S**NGGIYSIKKIINYPGK
EDVSVI**K**VEE**H**AI**K**RGPKGFNFNDNVTPFKYAAGAKAG**D**RIKVIGYPHPYKNKYVL**H**ESTGPVMSVEGSSIVYSA
HTESGNSGSPVLNSNNELVGIHFASDVKNDDNRNAYGVYFTPEIKKFIAENIDKGSWSHPQFEKGGGSGGGSGGS
SAWSHPQFEK

SplB variant B4:
ENNVTKVKDTNIFPYTGVVAFKSATGFVVGKNTILTNK**N**VSKNYKVGDRITAHPNSDKGNGGIYSIKKIINYPGK
EDVSVIQVEERAIERGPKGFNFNDNVTPFKYAAGAKAGERIKVIGYPHPYKNKYVLYESTGPVMSVEGSSIVYSA
HTESGNSGSPVLNSNNELVGIHFASDVKNDDNRNAYGVYFTPEIKKFIAENIDKGSWSHPQFEKGGGSGGGSGGS
SAWSHPQFEK

SplB variant B5:
ENNVTKVKDTNIFPYTGVVAFKSATGFVVGKNTILTNKHVSKNYKVGDRITAHPNSDKGNGGIYSIKKIINYPGK
EDVSVIQVEERAIERGPKGFNFNDNVTPFKYAAGAKAGERIKVIGYPHPYKNKYVLYESTGPVMSVEGSSIVYSA
HTESGNSG**P**PVLNSNNELVGIHFASDVKNDDNRNAYGVYFTPEIKKFIAENIDKGSWSHPQFEKGGGSGGGSGGS
SAWSHPQFEK

SplB variant B6:
ENNVTKVKDT**S**IFPYTGVV**LVN**SATGFVVGKNTILTNKHVSK**Y**YKVGDRI**N**AHPN**G**DK**A**NGGIYSIKKII**D**YPGK
EDVSVIQVEERAIERGP**N**G**Y**NFNDNVTPFKYAAGAKAG**D**RIKVIGYP**Q**P**H**KNKY**I**L**H**ESTGPVMSVEGSSIVYSA
HTESGNS**R**SPVLNSNNELVGIHFASDVK**D**DDNRNAYGVYFTPEIKKFIAENIDKGSWSHPQFEKGGGSGGGSGGS
SAWSHPQFEK

Target proteins

The target protein constructs contain an N-terminal 6xHis-tag (marked grey).

RickULP (GenBank accession number: ABV76659.1):
MAHHHHHHVGTQQQAPANNQKPWEKLGIPQEMYKESLKAEQQLAKPIIEPKQQIPEKKSSLVINTEDQVGVYNTG
NIKQPTYLYTEDDIKNILEANIDKNMFSIFHHASLEEPEILKDTLRVTVEDLILDNKPAIIPLNTGHKHWLLLMA
SKDDKGNINFMYNDPYGEPLESQPKVTEYITEIYPDAKITDLNTKQQANVYDCGVFVCDSAIKLSKGQKILTTEE
SKDQGINLRQAQANTLLIQQQAITIGHE

SseL (GenBank accession number: Q8ZNG2):
MAHHHHHHVGTMSDEALTLLFSAVENGDQNCIDLLCNLALRNDDLGHRVEKFLFDLFSGKRTGSSDIDKKINQAC
LVLHQIANNDITKDNTEWKKLHAPSRLLYMAGSATTDLSKKIGIAHKIMGDQFAQTDQEQVGVENLWCGARMLSS
DELAAATQGLVQESPLLSVNYPIGLIHPTTKENILSTQLLEKIAQSGLSHNEVFLVNTGDHWLLCLFYKLAEKIK
CLIFNTYYDLNENTKQEIIEAAKIAGISESDEVNFIEMNLQNNVPNGCGLFCYHTIQLLSNAGQNDPATTLREFA
ENFLTLSVEEQALFNTQTRRQIYEYSLQ

Synthetic peptide substrates used in activity assays

The substrate-specific activity assays were conducted using acetylated (Ac) tri- or tetra-peptides that are covalently linked to a 7-amino-4-methylcoumarin (AMC): Ac-X-AMC (X = Spl-specific peptide). They were either purchased from Peptanova (from the product catalog) or from Sigma Aldrich (Merck) through their custom peptide service. The substrates used in the study are listed in Table S3.

Table S3 Substrates used in the Spl-specific substrate assays. Substrates were always prepared freshly as a 25 µM working solution in the respective buffer. Ac = acetyl, AMC = 7-amino-4-methylcoumarin.

Abbreviation	Substrate (stock solutions stored at -20 °C)
Ac-YLY-AMC	Ac-L-tyrosyl-L-leucyl-L-tyrosyl-AMC (Sigma Aldrich); 10 mM in DMSO
Ac-VWLY-AMC	Ac-L-valyl-L-tryptophyl-L-leucyl-L-tyrosyl-AMC (Sigma Aldrich); 10 mM in DMSO
Ac-VEID-AMC	Ac-L-valyl-L-glutamyl-L-isoleucyl-L-aspartic acid-AMC (Peptanova); 10 mM in DMSO
Ac-WELQ-AMC	Ac-L-tryptophyl-L-glutamyl-L-leucyl-L-glutamine-AMC (Sigma Aldrich); 2.5 mM in 25% acetonitrile
Ac-LWLQ-AMC	Ac-L-leucyl-L-tryptophyl-L-leucyl-L-glutamine-AMC (Sigma Aldrich); 1.875 mM in 50% DMSO, 12.5% acetonitrile

Thermal stability of SplA and SplB WTs

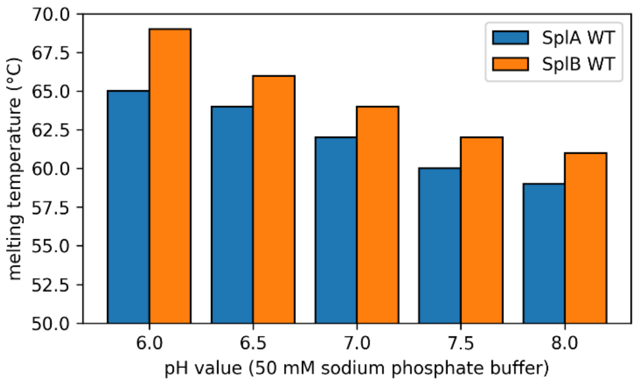

Figure S1 Thermal stability characterization of SplA and SplB WT. The melting temperatures of were determined in 50 mM sodium phosphate buffer at pH 6-8 by nano-differential scanning fluorimetry. A protein concentration of 1-2 mg/mL was used for the protein solutions in the measurements.

Michaelis-Menten kinetics of SplA allelic variants

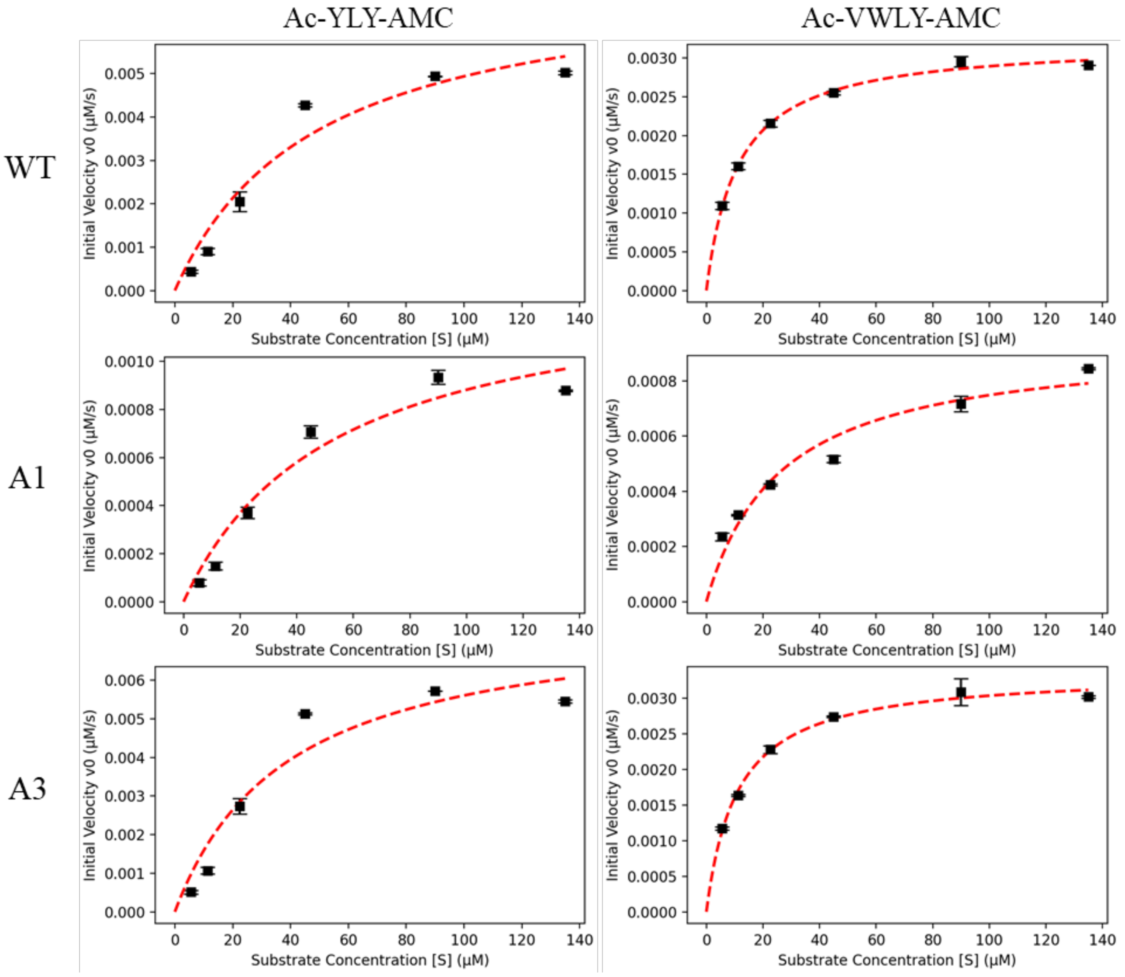

Figure S2 Enzyme kinetics of SplA WT and allelic variants A1 and A3 employing Ac-YLY-AMC and Ac-VWLY-AMC. Michaelis-Menten Fit (red dotted line) generated with “numpy.linspace”.

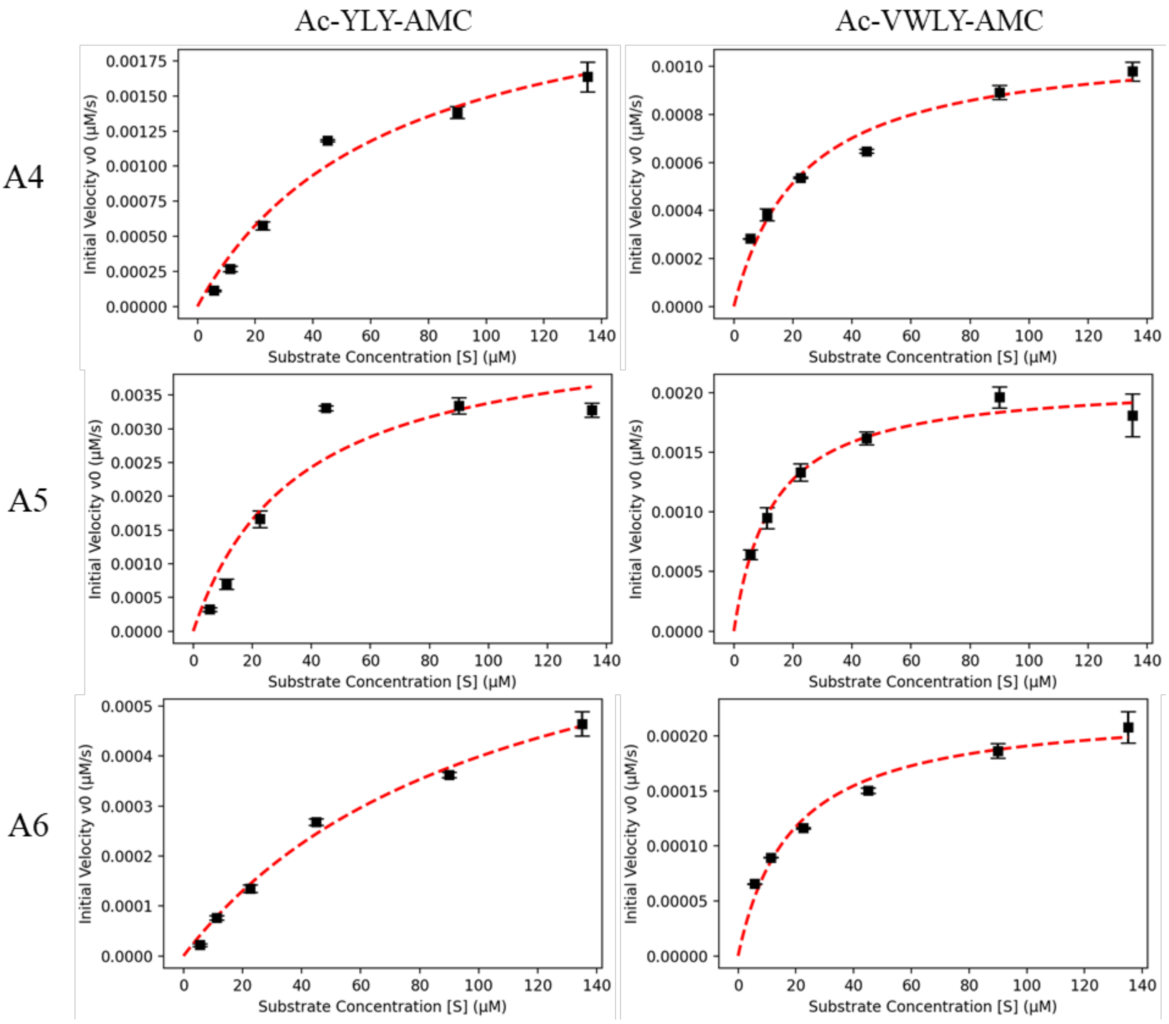

Figure S3 Enzyme kinetics of SplA WT and allelic variants A1 and A3 employing Ac-YLY-AMC and Ac-VWLY-AMC. Michaelis-Menten Fit (red dotted line) generated with “numpy.linspace”.

Initial substrate screening for SplA and SplB WTs

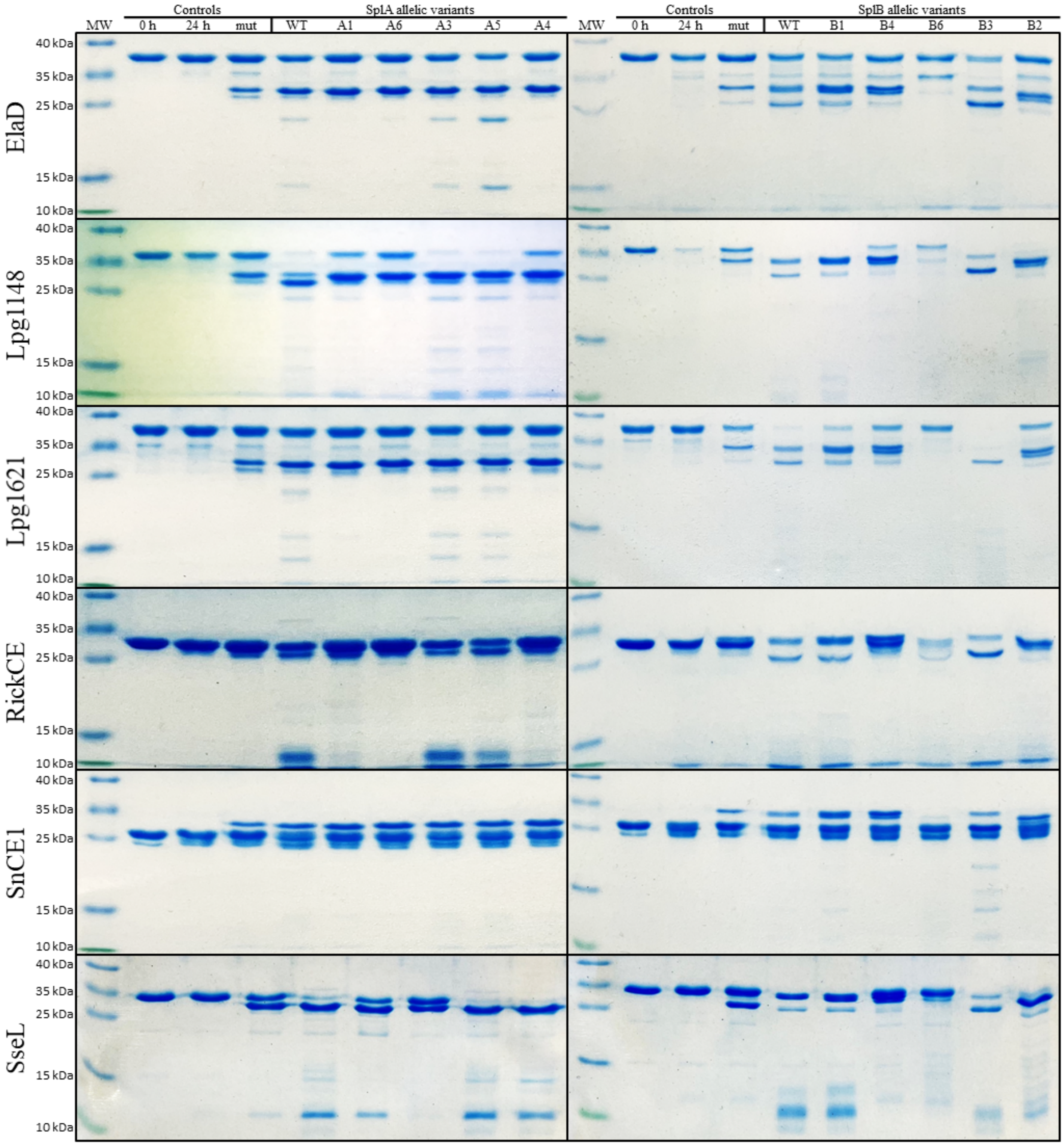

Figure S4 SDS-PAGE analysis of DUB/ULP cleavage by SplA and SplB allelic variants. Target proteins were incubated in a 2:1 ratio with SplA allelic variants in PBS and SplB allelic variants in 100 mM TRIS pH 8.2 at 37 °C for 24 h. Controls: target protein before (0 h) and after incubation in absence (24 h) or presence (mut) of inactive Spl mutants (with the catalytic serine exchanged to alanine: SplA_S154A, SplB_S157A). MW = molecular weight marker, contrast and brightness was adjusted for visual clarity.

Protein structure of Spl target protein RickULP

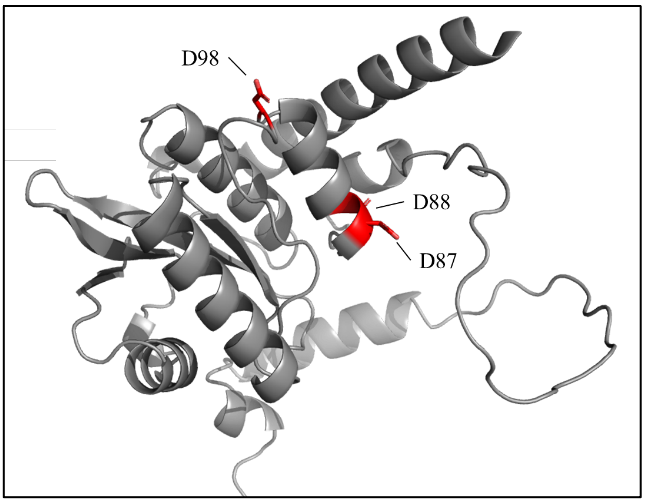

Figure S5 SplB-specific cleavage sites in RickULP. Predicted protein structure of RickULP (UniProt: A0A0H3AXY2) The aspartate residues investigated as potential SplB cleavage sites are marked in red. D98 is located on the protein surface and D87+D88 are occluded by a large loop structure.

Cleavage of RickULP mutants by SplA WT

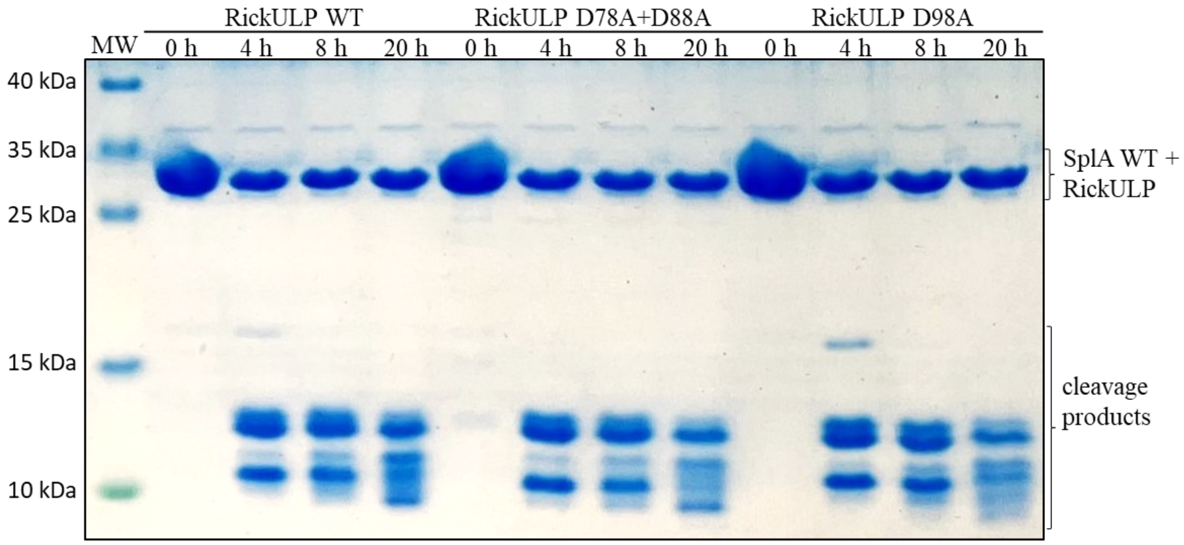

Figure S6 RickULP cleavage by SplA WT. SDS-PAGE analysis of RickULP WT, D87A+D88A double mutant and D98A single mutant cleavage by SplA WT. RickULP variants were incubated with Spls at 37 °C in 100 mM TRIS pH 8.2. MW = molecular weight marker, contrast and brightness have been adjusted for visual clarity.

Protein structure and electrostatic surface potential of Spls

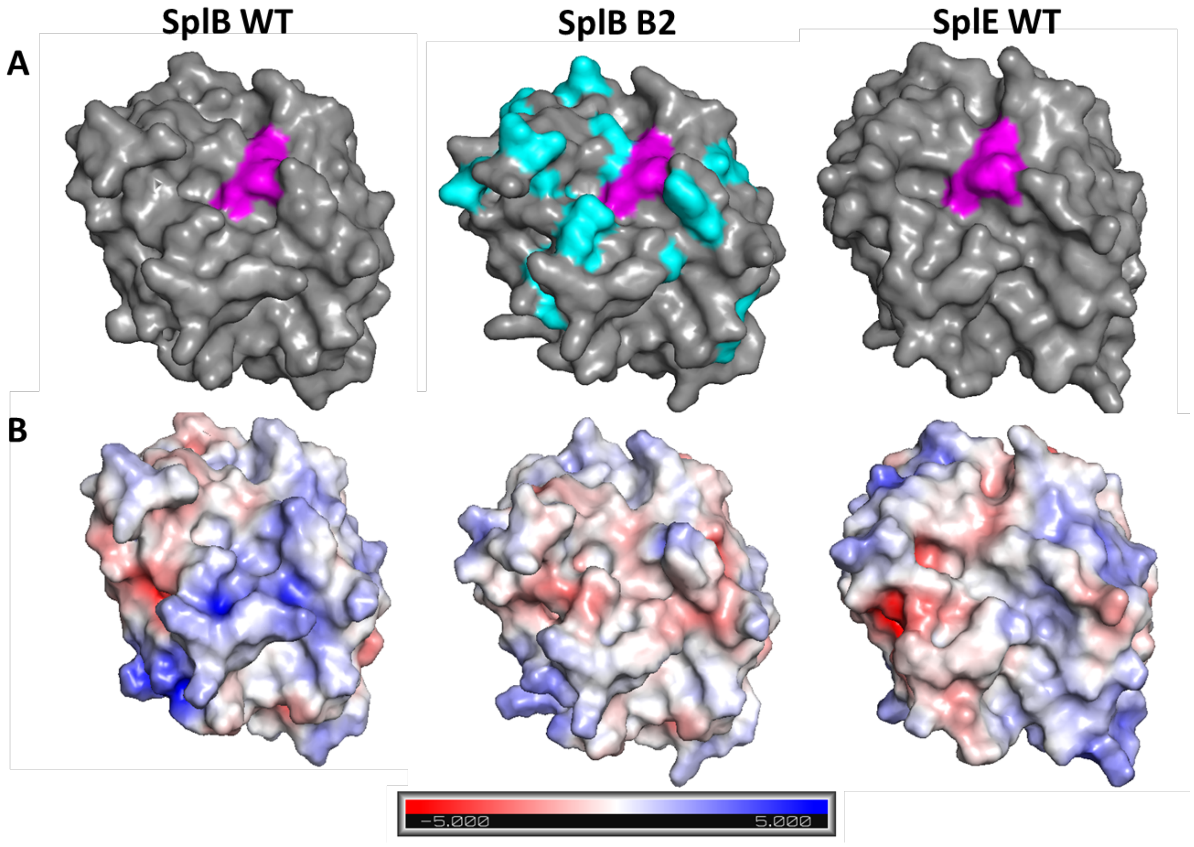

Figure S7 Protein surface of SplB WT, SplB allelic variant B2 and SplE WT. (A) Surface of Spls with the catalytic triad in magenta and the mutations in variant B2 compared to the SplB WT in cyan. (B) Electrostatic surface potential [k_BT/e_c] of Spls. Calculation for electrostatic surface potential was performed with the ABPS Electrostatics Plugin for PyMOL with 0.5 grid spacing. Proteins were prepared with the PDB2PQR method and the Connolly surface is displayed.

S. aureus lineages of investigated SpI variants

Table S4 Overview of *S. aureus* lineages for investigated SpI allelic variants. Clonal complex, methicillin-resistance and the composition of the *spl* operon (presence of *splA-F* genes) for each SpI variant. + = yes, - = no, +/- = can be both.

SpI variant	Clonal Complex	Methicilin-resistance	Genes contained in *spl* operon					
			splA	splB	splC	splD	splE	splF
**SplA WT**	8	+	+	+	+	+	+	+
**A1**	5	+	+	+	+	+	-	+
**A2**	5	+	+	+	+	+	-	+
**A3**	97	+	+	+	+	+	+	+
**A4**	121	-	+	+	+	-	-	+
**A5**	1	+	+	+	+	+	+	+
**A6**	8	+	+	+	+	+	+	+
**A7**	121	-	+	+	+	-	-	+
**SplB WT**	8	+	+	+	+	+	+	+
**B1**	5	+	+	+	+	+	-	+
**B2**	93	+	+	+	+	-	+	-
**B3**	121	-	+	+	+	-	-	+
**B4**	15	+	+	+	+	+	+	+
**B5**	15	+	+	+	+	+	+	+
**B6**	none	+/-	+	+	+	-	+	-

Contributor Roles

FG designed and performed experiments, analyzed the data, and wrote the draft manuscript. KM and LK performed experiments and analyzed the data. UB, DB and BB supervised FG, KM and LK. UB acquired funding and coordinated the project. OS and ML provided recombinantly produced proteins. TJ prepared and provided datasets. CH, LS and UV performed MS-measurements and analyzed data. All authors reviewed and approved the manuscript
